# Supplementary material for: Female Brain and Endocrinological Research–Veteran (FemBER-Vet) study: A study protocol for identifying endocrinological, lifestyle and psychosocial determinants of brain health outcomes in female veterans for future intervention success
Source: PLoS One. 2025 Jan 22;20(1):e0306149. doi: 10.1371/journal.pone.0306149 (PMC11753646; doi:10.1371/journal.pone.0306149)
Supplement: Attachment — Submitted filename: RESPONSE.docx [file pone.0306149.s001.docx]

Dear Dr Zimmerman,

**RE: PLOS ONE Decision: Revision required [PONE-D-24-18322] - [EMID:b6c6dd189fed26f4]**

Thank you for your further comments, please see responses below, in turn.

1) The file called "manuscript" is the original manuscript, not the revised ms. Please replace this with an unmarked version of your revised paper without tracked changes. You should upload this as a separate file labeled 'Manuscript'.

**Apologies, I did not realise I also need to provide a clean version again at this stage, I have updated this as a clean version now.**

2) The file called "Revised Manuscript with Track Changes" does appear to be a revised manuscript, but (a) there are no track changes, and (b) "female" is still used as a noun in several places. Please replace this with a marked-up copy of your manuscript that highlights changes made to the original version. You should upload this as a separate file labeled 'Revised Manuscript with Track Changes'.

**I am not sure why you cannot see track changes, but I’ve run some stress testing and believe this is an artifact of your “build your PDF” system, as twice I attempted to do this with the corrected document, even highlighting these in yellow so they are more obvious and the “Build your PDF” product did not show these. I have now created my own PDF of the tracked changes manuscript and uploaded this. Its appears the final PDF for approval now contains these.**

**As with regards to language, I have updated the manuscript in accordance with your preference as much as possible. I have also reviewed the new updated manuscript and run it through AI to identify places where updating of nouns to woman/women are still outstanding. I believe this is now complete as much as possible. However, if you are referring to references such as “ female military veterans” in stead of “women military veterans” where it is being used as a gerund, I am afraid that I am reluctant to update this to the latter. We work closely with our military and LGBTQIA+ stakeholders surrounding language for this project. The draft was subject to review for language on gender by LBTQIA+ pilot military and civilian participants and the term female and male as nouns were chosen to designate sex assigned at birth reference *by them*. Nonetheless, I have made these changes as you have asked. This preference was shown also by non-pilot participants but who were two members of the trans community. Respectfully, we write for, and are dedicated to upholding the preferences of, our stakeholders and I would be reluctant to make further changes to the gerunds as described above. It is difficult to anticipate all preferences, but I would suggest that the APA guidance is not relevant for local North-East of UK preferences at least amongst our LGBTQIA+ and military stakeholders.**

Sincerely,

Tam

Dr Tamlyn Watermeyer

- Apologies for lac of headed paper – I am away at a conference and working internationally and it is difficult to gain necessary stationery, even via online means.
